# Fused Filament Fabrication of Polymers and Continuous Fiber-Reinforced Polymer Composites: Advances in Structure Optimization and Health Monitoring

**DOI:** 10.3390/polym13050789

**Published:** 2021-03-04

**Authors:** Fatemeh Mashayekhi, Julien Bardon, Vincent Berthé, Henri Perrin, Stephan Westermann, Frédéric Addiego

**Affiliations:** Department Materials Research and Technology (MRT), Luxembourg Institute of Science and Technology (LIST), ZAE Robert Steichen, 5 Rue Bommel, L-4940 Hautcharage, Luxembourg; fatemeh.mashayekhi@list.lu (F.M.); julien.bardon@list.lu (J.B.); vincent.berthe@list.lu (V.B.); henri.perrin@list.lu (H.P.); stephan.westermann@list.lu (S.W.)

**Keywords:** fused filament fabrication, thermoplastic polymer, continuous fiber-reinforced thermoplastic composites, structure, interface, sensor, technology landscape

## Abstract

3D printed neat thermoplastic polymers (TPs) and continuous fiber-reinforced thermoplastic composites (CFRTPCs) by fused filament fabrication (FFF) are becoming attractive materials for numerous applications. However, the structure of these materials exhibits interfaces at different scales, engendering non-optimal mechanical properties. The first part of the review presents a description of these interfaces and highlights the different strategies to improve interfacial bonding. The actual knowledge on the structural aspects of the thermoplastic matrix is also summarized in this contribution with a focus on crystallization and orientation. The research to be tackled to further improve the structural properties of the 3D printed materials is identified. The second part of the review provides an overview of structural health monitoring technologies relying on the use of fiber Bragg grating sensors, strain gauge sensors and self-sensing. After a brief discussion on these three technologies, the needed research to further stimulate the development of FFF is identified. Finally, in the third part of this contribution the technology landscape of FFF processes for CFRTPCs is provided, including the future trends.

## 1. Introduction

Continuous fiber-reinforced thermoplastic composites (CFRTPCs) have gained considerable attention in automotive and aeronautics applications due to their attractive strength-to-weight ratio, enabling energy and recyclability to be saved as suitable end-of-life scenario. The development of fused filament fabrication (FFF) by 3D printing technology enables us to reduce lead time, accelerate design iteration, and fabricate complex shape-eliminating sub-components [1]. The quest for printed parts with high mechanical performance led to the development of innovative FFF processes where continuous fibers are incorporated into printed structures [2,3]. The different thermoplastic polymer (TP) matrices and CFRTPCs that can be FFF-printed are reported in [4,5], including their process variables and the resulting tensile strength. For example, the tensile strength of neat TP ranges from 34 MPa in the case of acrylonitrile butadiene styrene (ABS) to 100 MPa in the case of polyether ether ketone (PEEK). Concerning CFRTPCs, their tensile strength can reach several hundreds of MPa depending on the composite design, fiber content, and fiber orientation, approaching that of aluminum under certain conditions [6]. 3D printed CFRTPCs appear very attractive, but the scale-up of this promising technology from the prototyping to the production of finished functional components is not straightforward [4]. This may be attributed to the long build time and non-optimal mechanical properties of the 3D printed materials, as highlighted in [7]. To address these issues, a better understanding of the rheological behavior of the TP may be of great help since FFF involves heat transfer from the extruder barrel to the TP, extrusion of the melted TP, shear and elongation of the TP when entering the nozzle, and die swell of the TP when exiting the nozzle [7,8,9]. The rheological behavior of the TP is also expected to be drastically influenced by the presence of the continuous fibers during the printing process. In this review, we do not discuss the recent advances in the rheology of the 3D printed materials, and rather point the readers to an interesting review on this topic [7]. Instead, we focus on polymer and composite science and engineering approaches to understand and optimize the mechanical properties of the 3D printed materials. 

**Figure 1:**
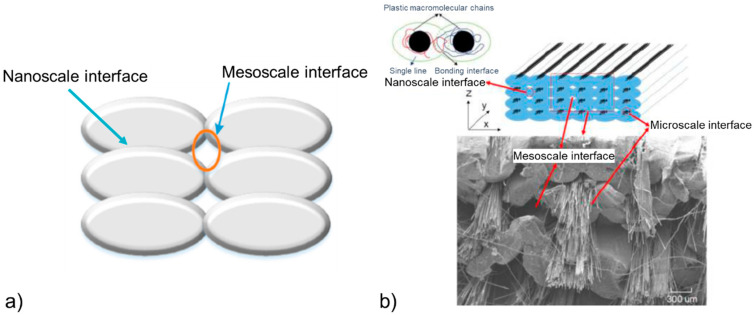
Multiscale interfaces in 3D printed (**a**) neat TPs [5] and (**b**) CFRTPCs [10] (copyright (2017), with permission from Emerald Publishing Limited). Nanoscale and mesoscale interfaces are generated between two and four printing lines, respectively, and microscale interfaces are generated between the TP matrix and the fiber bundle.

**Figure 2:**
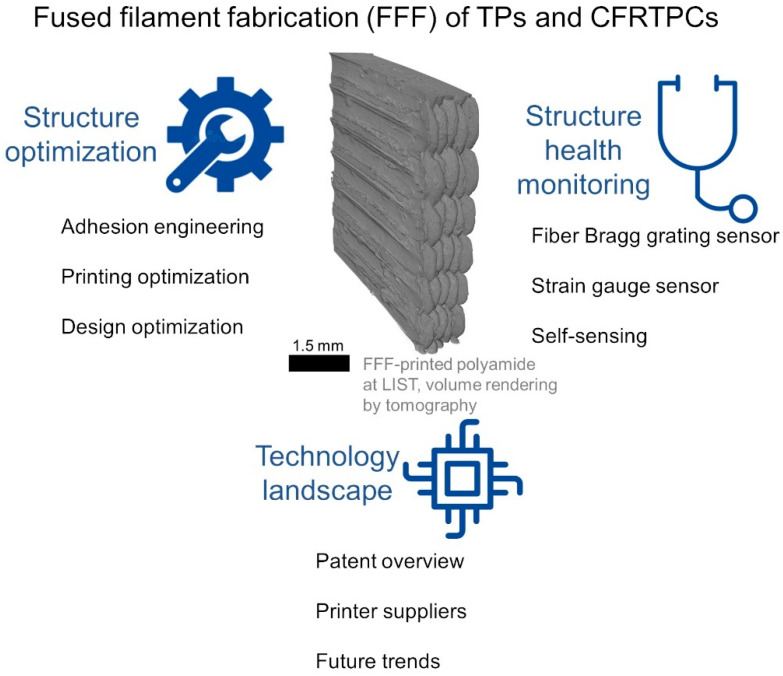
Scope of the present review on FFF-printing of TPs and CFRTPCs. Three pillars are addressed: structure optimization, structure health monitoring, and technology landscape.

The FFF of neat TPs and CFRTPCs leads to structures with interfaces at different scales that are detrimental for the mechanical properties when compared to the same materials manufactured by traditional processes employing high pressures. For example, FFF-printed ABS reinforced with continuous carbon fibers exhibits a tensile strength of about 150 MPa, which is 33% lower than that of the same composite processed by injection molding [10]. This result can be explained by the presence of three types of interface in the FFF-printed materials engendering non-optimal mechanical properties [5,10,11,12]. First, the literature reports the existence of nanoscale interfaces between two filling lines (also called weld lines), involving a non-optimal polymer interchain diffusion. Second, FFF is characterized by microscale interfaces between the impregnated fiber bundle (or towpreg) and the polymer matrix, linked to poor polymer-fiber adhesion or wettability. Last, mesoscale interfaces between each of the four filling lines with potentially a lack of bonding have been highlighted. These interfaces are represented in Figure 1a in the case of neat TPs and in Figure 1b in the case of CFRTPCs. The high attractiveness of 3D printed materials has led to a significant number of research studies within the last 10 years aiming at improving the bonding at these different interfaces. The first part of this review provides a detailed description of these three interfaces and explores the most efficient methods to improve the interfacial bonding based on adhesion engineering, printing optimization, and material design optimization (Figure 2). 

If mechanical properties are of primary interest for structural applications requiring load-bearing capacity, the implementation of other functionalities can further increase FFF-printed materials’ attractivity and enlarge their application range. To this end, numerous functionalities have been developed as sensing, self-healing, magnetic properties; thermal properties; and electrical properties [13]. Among those functionalities, the possibility to monitor the structural health is of high interest to reduce maintenance costs, increase reliability, optimize security, and improve material design [14]. In the second part of this review, the last developments in the field of health monitoring of 3D printed materials are presented with a focus on strain sensors to detect damage, including fiber Bragg grating (FBG) sensors, self-sensing approaches, and strain gauge sensors. The advantages and drawbacks of these sensors are discussed (Figure 2). 

Despite the limited number of research papers on FFF technology, this very recent and growing domain proves to be quite innovative and active. Our objective in the last part of the review is to address the applied aspects of those developments by drawing the current technology landscape of FFF processes for CFRTPCs. The current suppliers of FFF systems enabling CFRTPCs to be printed and the main characteristics of those systems are briefly described in this part. Finally, the future trends of this technology are identified (Figure 2). 

## 2. Structure Optimization 

### 2.1. Physical Description of the Interfaces

**Figure 3:**
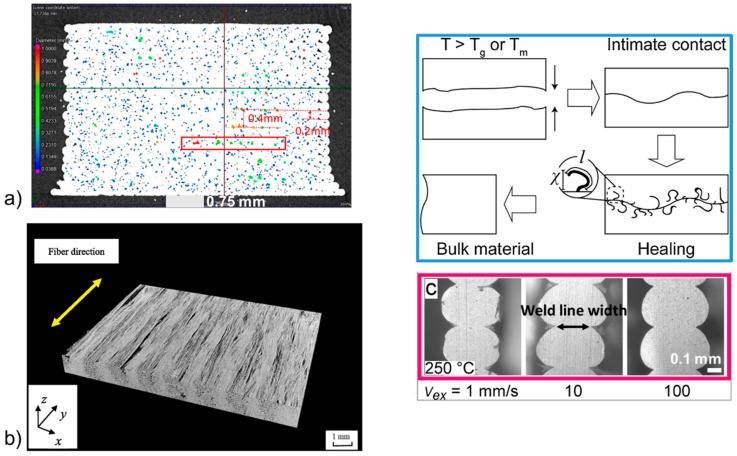
Visualization of interfaces in 3D printed materials: (**a**) mesoscale interfaces between printing lines in neat PLA by µCT analysis [17], (**b**) microscale interfaces resulting from fiber bundle-matrix debonding in continuous carbon fiber-reinforced PA observed by µCT [18] (copyright (2020), with permission from Elsevier), and (**c**) nanoscale interfaces in neat acrylonitrile butadiene styrene (ABS) observed by optical microscopy from thin sample slices, including the principle of this interface formation [24,27] (copyright (2017) and (2019), with permission from Elsevier).

Interfaces in 3D printed composites were visualized by different methods. Micro-computed X-ray tomography (µCT) was proven to quantify mesoscale and microscale interface porosities in 3D [15,16,17]. It was shown that in 3D printed polylactide (PLA), the pore volume fraction was in the range of 4–7 vol% (depending on printing design) and that 99% of the pores between printing lines were in the size range between 0.03 and 0.2 mm and 1% were in the size range between 0.2 and 1 mm (Figure 3a). 3D printed CFRTPCs were also analyzed by µCT [15,16,18]. For example, in the case of polyamide (PA) reinforced with continuous carbon fibers, mainly microscale interfaces were observed engendering pores attaining a thickness of 300 µm and a volume fraction of 10% (Figure 3b) [18]. In addition to pores induced by debonding between fiber bundles and the polymer matrix, other defects were detected by µCT as fiber bundle disbonding and fiber damaging [15]. 

Electron microscopy techniques have been widely used to characterize in 2D mesoscale and microscale interfaces with a higher resolution compared to µCT [19,20,21]. However, only 2D information is provided and to observe samples prior to any mechanical testing, careful sample preparation is required to avoid the induction of defects due to the preparation (water-jet cutting [20], ion abrasion methods [22]). In particular, pores of several microns resulting from the release of gas were identified in CFRTPC printing lines [11]. 

Concerning nanoscale interfaces (weld line), direct observation can be conducted by electron scanning electron microscopy [23] and optical microscope [24]. The weld line width in the case of acrylonitrile butadiene styrene (ABS) was measured to be in the range of 170–300 µm (nominal line width of around 500 µm) (Figure 3c), depending on printing speed and temperature [24]. Concerning weld line thickness or interpenetration depth, it is dictated by the polymer interchain diffusion mechanisms at the interface. These mechanisms have been investigated in detail based on models in the paper by [25]. One important finding was that the interpenetration depth was around two times the radius of gyration (R_g_) of the polymer, meaning around 20 nm for PA [26]. However, as highlighted in Figure 3c, the intimate contact between the two polymer faces is not perfectly flat and besides, the nozzle may contain impurities that interact with the polymer at the molten state. It results that practically, the weld line thickness is probably higher than 2R_g_, its exact thickness being not easily measurable. 

### 2.2. Mechanical Properties

**Figure 4:**
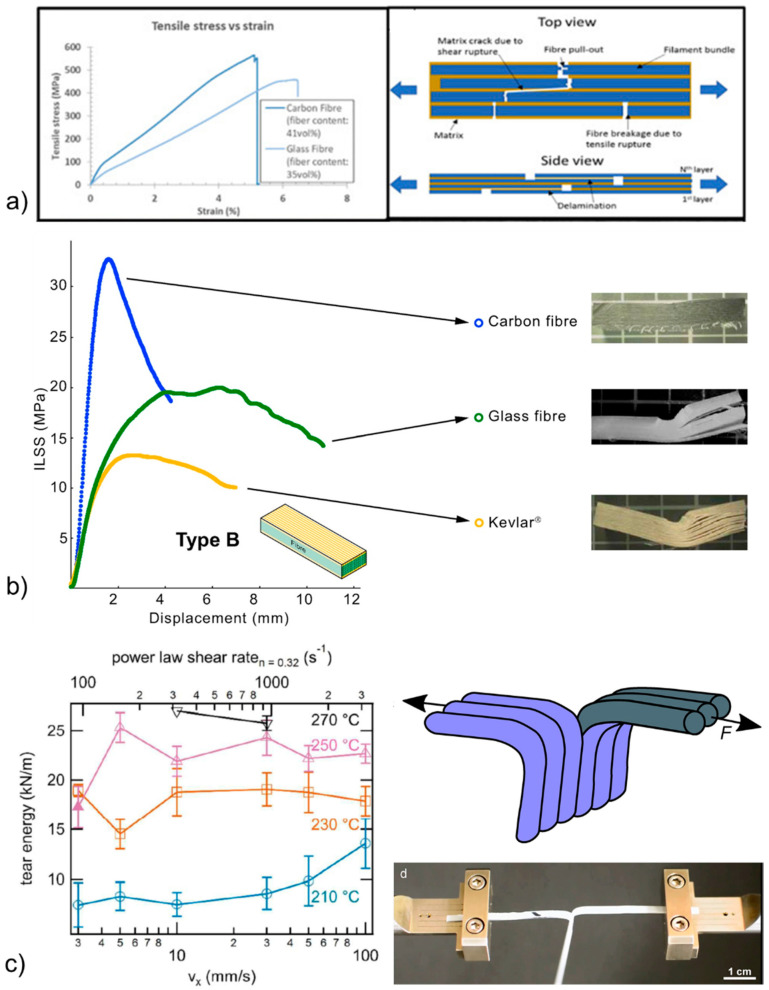
Main mechanical testing of 3D printed neat TPs and CFRTPCs: (**a**) tensile testing of 0° UD laminate composite (PA with carbon or glass fibers) [29] (copyright (2018), with permission from Elsevier), (**b**) short beam shear (SBS) testing to measure the interlaminar shear strength (ILSS) of glass and Kevlar fiber-reinforced PA, [31] (copyright (2018), with permission from Elsevier), and (**c**) welding line adhesion measured by “trouser tear” Mode III fracture testing in the case of neat acrylonitrile butadiene styrene (ABS) [24,33] (copyright (2017), with permission from the Royal Society of Chemistry and Elsevier).

Tensile testing on the laminate of unidirectional plies (UD) (0° or 90°) has been widely considered to evaluate the tensile modulus and the tensile strength of 3D printed composites. In general, tensile testing conducted in the case of 0° UD composites (along printing line/fiber direction) exhibited an elongation at the break in the range of 1–5% of the strain (Figure 4a), whereas the tensile modulus and strength varied significantly from one study to another study, depending on the composite design (tensile modulus in the range of 2.4–61 GPa, tensile strength in the range of 27–700 MPa) [28,29,30]. Compared to CFRTPCs fabricated by conventional methods, and for a similar fraction of fibers, 3D printed CFRTPCs exhibited a similar or even higher tensile strength, but an elastic modulus four to five times lower, as shown in the case of carbon fiber-reinforced PA [29]. This lower elastic modulus can be attributed to mesoscale and microscale interfaces exhibiting pores, and to the presence of nanoscale interfaces (weld lines). In the case of 3D printed neat TP (PLA), the highest tensile modulus and strength were obtained for the material exhibiting the lowest porosity fraction [17]. Accordingly, the presence of pores is currently being introduced in the model predicting the elastic modulus of 3D printed TP [17]. The tensile properties for 90° UD composites are obviously lower than for 0° UD composites, the three different kinds of interfaces being highly solicited [28,30]. One limitation of such tensile testing is that it provides the overall macroscopic mechanical signature of the printed structure, including the deformation signature of the matrix to a certain extent, and the failure signature of all the interfaces, as represented in Figure 4a. To improve the material, it is important to reveal the influence of its weakest points on mechanical properties and/or to reveal the intrinsic behavior of the TP matrix. 

Accordingly, interlaminar testing was developed to specifically stress the weakest points of the structure being the three interfaces (Figure 1). To this end, short beam shear (SBS) testing was applied to 3D printed materials to measure the maximum amount of shear that can withstand the laminate between layers before failure. In principle, for carbon fiber-reinforced TP, SBS generated a crushing of the carbon fibers at the sample side in compression. This phenomenon induced a crack that propagated to the opposite side of the sample that was in tension, soliciting mesoscale and nanoscale interfaces of the printed lines [29]. SBS testing enables the interlaminar shear strength (ILSS) to be extracted, as represented in Figure 4b [19,31]. In the case of neat PA printed in 3D, ILSS slightly decreased (from 10.2 MPa to 9.3 MPa) as the printed line thickness increased (from 0.1 mm to 0.2 mm) due to a higher porosity content [31]. In the case of 3D printed PA composites, ILSS decreased in this material order: PA/carbon fibers (22.2 MPa for 27 vol% of fibers) > PA/glass fibers > PA/kevlar fibers. This finding was related to the fibers-matrix wettability that was the highest for carbon fibers and the worst in the case of Kevlar, showing the influence of microscale interfaces [31]. 

Recently, interlaminar fracture under mode I loading was successfully applied to CFRTPCs based on double cantilever beam (DCB) testing [32]. For this testing, a pre-crack is introduced in the 3D printed composite with an insert. This measurement provides the energy dissipated by the formation of new fracture surfaces from the initial crack propagation. However, the printing of the samples appears very complex. As for nanoscale interfaces, specific methods have been developed to relate printing conditions (temperature and speed) and polymer viscosity to the nanoscale interfacial adhesion in neat 3D printed TP [24,27,33]. This interfacial adhesion influenced by the interpenetration depth [25] was measured by “trouser tear” Mode III fracture testing (Figure 4c) [24] and by the ratio between the tensile strength of the printed interface to bulk tensile strength based on chain reptation theory [27]. It was demonstrated that nanoscale interfacial adhesion increased with printing temperature and with decreasing polymer viscosity by adding a plasticizer, whereas printing speed had a limited influence on this interfacial adhesion (Figure 4c). 

### 2.3. Improving Interfacial Bonding

Numerous methods are currently in development to increase neat TP and CFRTPC interfacial bonding, as detailed in a previous review [34]. The main approaches can be classified into three categories: (1) adhesion engineering, (2) printing quality optimization, and (3) material design optimization. 

#### 2.3.1. Adhesion Engineering 

**Figure 5:**
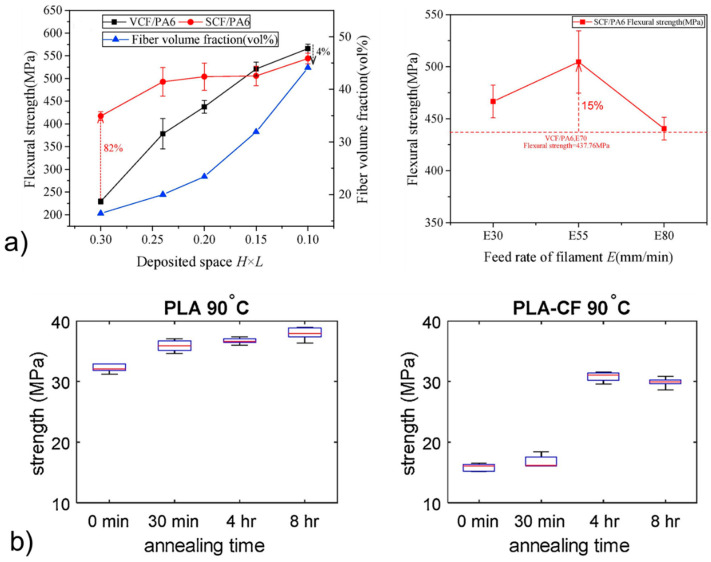
(**a**) Effect of printing parameters, sizing, and fiber fraction on the mechanical strength of 3D printed PA composite [19] (copyright (2018), with permission from Elsevier) and (**b**) effect of annealing on the mechanical strength of 3D printed PLA [20] (copyright (2019), with permission from Elsevier).

Adhesion engineering methods aim at improving the bonding at the continuous fiber-matrix interface and between printed lines, thereby enhancing adhesion at the microscale and mesoscale interfaces. Several approaches were considered, including the use of a coupling technology between the carbon fibers (generally impregnated with epoxy) and the TP matrix [19,21]. It was proven that an appropriate carbon fiber sizing has to be developed when considering a TP as matrix [19]. When comparing unsized and sized carbon fiber, the flexural strength of PA composite increased by 82% at a fiber volume fraction of 16% (Figure 5a). The sizing consisted of removing the epoxy impregnation with acetone and then dipping the unsized fiber in a water-based solution of PA. Last, the PA sizing was melted through the printed nozzle to homogenize it. In addition, the effect of a plasma pre-treatment of carbon fibers before their impregnation in a polyether-ether-ketone (PEEK) TP matrix was investigated [35]. It was shown that not only was the interlayer bonding improved but also the stability of the printing process. Another relevant method found in the literature is the use of adhesion promoter, as polydopamine (PDA) that was successfully used in the 3D printing process of PLA [36]. In particular, PLA pellets were coated with PDA prior to extrusion, resulting in a 10% increase in tensile strength compared to untreated PLA. Some structural analysis by X-ray photoelectron spectroscopy and Fourier-transform infrared spectroscopy proved a covalent immobilization of PDA onto PLA. Note that this methodology, inspired by mussel adhesive proteins [37], was previously used in the case of epoxy reinforced with continuous carbon fibers manufactured by conventional techniques. In this case, there was an increase of 20–25% of fracture toughness by DCB testing and of ILSS compared to the untreated composite, due to the increased bonding between epoxy and carbon fibers [38]. PDA was also successfully applied in TP reinforced with carbon fiber based on a self-assembly approach to treat carbon fibers [39]. In the case of neat 3D printed TP, epoxy adhesive was used to improve the flexural strength of the material [40]. However, a specific process has been developed to have an effective influence of the epoxy. ABS was printed with channels oriented perpendicular to the interlayer direction, and the plasma surface was treated to increase wettability and infiltrated with an epoxy/hardener mixture. The flexural strength was demonstrated to improve by 50% with the plasma treatment and by 130% when combining plasma treatment and the epoxy adhesive treatment. One limitation of this method is the long treatment time needed to fully cure the epoxy resin. 

#### 2.3.2. Printing Quality Optimization 

Then, many approaches were proposed to improve the quality of FFF printed parts. They generally aimed at improving interlayer bonding through better management of both mesoscale interfaces and nanoscale interfaces. For the latter, several strategies targeting better polymer interchain diffusion at the interlayer level were considered. They are based on optimizing printing parameters and applying various treatments, including treatments of the filament and/or interlayer interface and post-treatments of the 3D printed material. 

It was first shown that printing parameters should be optimized concerning mechanical properties [19,23,27,33,41,42]. For example, the flexural strength of 3D printed PA6 composites had an optimal value concerning filament feeding rate (Figure 5a) [19], and the tear energy increased with the nozzle temperature in the case of neat ABS (Figure 4c) [24]. Note that the flexural strength increased with the fraction of fiber, as represented in Figure 5a for PA composites [19]. The critical role of polymer interchain diffusion for the management of interlayer bonding is mentioned in several studies [27,42]. 

**Figure 6:**
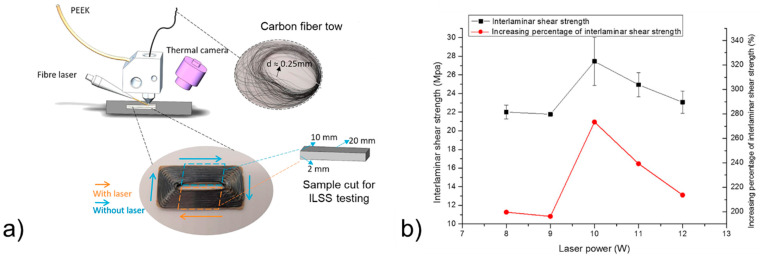
Laser-assisted printing process [21]: (**a**) diagram of the preparation of continuous CF/PEEK composite samples for ILSS testing and (**b**) ILSS and increasing percentage of ILSS with different laser powers (copyright (2019) with permission from Elsevier).

The printing process can be modified by introducing an in situ pre-heating of the base layer before the successive weld line is deposited. This thermal pre-treatment aims at improving nanoscale interface bonding by promoting interchain diffusion. The treatment can be conducted with a laser [21,43], an infrared lamp [44], or forced hot air [45]. In the case of laser pre-heating, the optimal temperature was found to be between the glass transition temperature and the degradation temperature of PEEK (Figure 6a). Under these conditions, ILSS increased by a factor of three (Figure 6b). The coupling of two approaches aiming at improving different interfaces was implemented by the same research team through the development of a plasma pre-treatment of carbon fibers (CF) before impregnation of the fibers by PEEK [35]. Then CF pre-impregnated filament was printed in laser-assisted FFF equipment to make samples for ILSS testing. It was reported that the implementation of both plasma and laser treatments in the overall printing process provides the best samples in terms of flexural properties and reduced porosity. 

Advances in chemical modifications of filaments or interfaces to improve the quality of printing are discussed in the literature. For instance, Ko et al. [27] investigated the addition of small amounts of triphenyl phosphate (TPP) plasticizer in a polycarbonate (PC)-ABS blend filament. It was reported that this addition of plasticizer improved the interfacial bonding of printed parts through an improved degree of healing. In another study [46], benzene-derivate plasticizers obtained from the pyrolysis of trisilanolphenyl polyhedral oligomeric silsequioxane (POSS) were successfully used to improve the mesoscale interface bonding of 3D printed PEEK. In addition, a method based on atmospheric pressure cold plasma treatment of the polymer filament is under development [47]. More precisely, an atmospheric pressure dielectric barrier discharge (DBD) plasma torch has been implemented on a 3D printer. The objective of this technology is to modify the wettability of the base printed line to better deposit the welding line onto it, enhancing polymer adhesion by introducing new polar groups at the treated polymer surface. Some initial promising results were obtained on the influence of this plasma treatment on the interfacial adhesion, but a deeper knowledge on the influence on the treatment parameters (voltage, power, gas composition and flow rate, exposure time, etc.) on the efficiency of the plasma treatment is required. Another approach relies on the use of low-molecular-weight surface-segregating additives in the polymer filament that can diffuse and entangle in adjacent lines during the printing [48,49]. These linear additives can also contain a functional group providing a three-arm structure, reacting with UV irradiation that was applied in situ during the printing. This functionality led to a covalent bonding between adjacent additive molecules. An increase in the transverse maximum tensile stress of FFF printed PLA of 140% in the case of linear additive and of 200% in the case of the three-arm additive was noted compared to as-printed PLA. It was discussed that a balance has to be found between the covalent bonding of the additives and the mobility of the latter to diffuse across the mesoscale interface. In [50], neat PLA was blended with a radiation sensitizer prior to 3D printing. After FFF, specimens were irradiated with gamma rays (50 kGy, 60 °C) and tested. The mechanical testing showed a 50% increase in tensile strength when applying strain perpendicular to the printed lines This finding was explained by the crosslinking reaction between printed lines, however, in this previous study, the stability of the irradiated specimens (presence of free radicals) was not studied. 

Different kinds of thermal post-treatments of the 3D printed material were investigated. This post-treatment can be performed by a simple annealing procedure [20,51], a combination of annealing and pressure [52], annealing in the presence of thermally expandable microspheres [53], microwaves [54], or ultrasonic vibration [55]. In the case of microwaves post-treatment, a special carbon nanotube ink was developed to cover the TP filament in the printer [54]. This ink is used as a heating source when irradiated with microwaves to further improve the polymer interchain diffusion between the printed lines, providing a 275% increase in the fracture strength (“trouser tear” Mode III fracture testing) compared to the untreated 3D printed materials. Moreover, the ability of this design to locally induce heating through this microwave-sensitive coating allows the annealing treatment—as low as 60 s of microwave treatment—to be sped up by heating the part where it is necessary, i.e., close to the interfilament interfaces. Concerning annealing, in the case of a semi-crystalline TP, the optimal temperature is between the glass transition temperature and the cold crystallization temperature. Indeed, the formation of crystals may hinder the interchain diffusion [20]. In the case of PLA, some cold-crystallization investigations by X-ray diffraction (XRD) revealed that PLA started to crystallize quickly from 90 °C, and that decreasing viscosity (by mechanical recycling) increased its crystallization rate [56]. This temperature was chosen as the post-treatment annealing temperature in the case of 3D printed neat and reinforced PLA with short fibers, for which tensile strength increased by around 20% for neat PLA and by around 100% for the PLA composite compared to untreated materials (Figure 5b). 

#### 2.3.3. Material Design Optimization

Another approach based on the material design optimization can be considered to obtain printed parts with better mechanical properties. This approach involves the screening of the raw material properties to detect risks and the reduction of the printed material anisotropy. 

In the works of [57], screening the towpreg and the polymer matrix structural properties prior to printing was proposed to potentially identify any risks that may be detrimental for the interfacial bonding. Accordingly, the composition, microstructure, and mechanical properties of these two constituents were carefully analyzed. The main findings can be summarized as follows: (1) Depending on its nature the polymer matrix can absorb up to 8% of water and needs to be dried before printing to avoid bubble and pores [11]; (2) the polymer resin used for impregnating the fibers and the TP polymer composite matrix were often of different nature and microstructure, influencing interfacial bonding and the modelling material inputs; and (3) the carbon towpreg may exhibit a heterogeneous spatial distribution of its two constituents (carbon fibers and the impregnating resin), drastically influencing the stress distribution and hence, the mechanical performance. Therefore, any identified risks should be addressed prior to printing. 

Last, advances in the 3D printing design of polymers [58] or polymer composites [59] were proposed based on the build direction. The first study disclosed the implementation of the so-called Z-pinning to reinforce the mechanical properties of printed parts in the Z (perpendicular to layers) direction. The second study was inspired by designs already developed for traditional composites in which reinforcement fibers are generally oriented in the three directions of the composite parts. It consequently proposed printing CFRTPCs along sinusoidal trajectories. The interest of this novel concept to reduce mechanical anisotropy was demonstrated by modifying the printing trajectories on the XY plane (layer axis). As an outlook, extending the concept in the Z (perpendicular to layer) direction is proposed.

### 2.4. Molecular Behavior of the Matrix 

**Figure 7:**
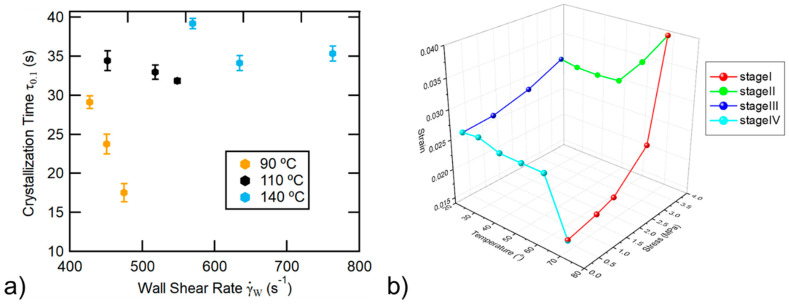
Thermal and mechanical molecular behavior of 3D printed TP: (**a**) crystallization kinetics of PCL as a function of nozzle wall shear rate and temperature [60] (copyright (2018), with permission from Elsevier), and (**b**) amorphous chain orientation mechanisms of PLA nanocomposite upon cyclic stress and thermal solicitations (increase in strain = decrease in distance between chain, and inversely) (stage I: stress loading at 70 °C, stage II: cooling down to 22 °C at the maximum stress level, stage III: stress unloading at 22 °C, and stage IV: heating up to 70 °C without stress) [67] (copyright (2019), with permission from Elsevier).

Knowledge of TP crystallization kinetics may help to find the best printing conditions for improving interchain diffusion at the nanoscale interface, and in general, may contribute to understanding the long-term stability of the material. In the case of 3D printed TP, they were studied in two pioneer studies [60,61]. In particular, a special method combining infrared thermography and Raman spectroscopy was developed to measure the temperature and the crystallinity during the extrusion of polycaprolactone (PCL). Note that as highlighted in another work, the cooling rate during the deposition of the melted polymer onto the previous layers at the substrate temperature is in the range of 20–50 °C/s [62]. In [60], measurements were taken at the surface of an extruded line of PCL and it was revealed that crystallization is faster when decreasing the extrusion temperature and increasing the nozzle wall shear rate (Figure 7a). It was explained that increasing the shear rate facilitated flow-induced crystallization. However, as highlighted by the authors, a skin/core effect of the extruded line was expected with skin more crystallized than the core due to higher shear at the outer surface. This study highlighted the structural complexity of the extruded line of TP. Obviously, this extrusion-induced anisotropic structure of the TP matrix is expected to influence both crystallization kinetics and chain-orientation mechanisms upon an applied mechanical strain. 

Chain orientation mechanisms were analyzed by small- and wide-angle X-ray scattering (SAXS/WAXS), as commonly done for semi-crystalline TP processed by conventional extrusion or compression molding [63,64,65,66]. SAXS and WAXS enable amorphous chain orientation, crystalline plan orientation, crystalline phase transformation, crystalline lamellae orientation, and strain-induced cavitation to be characterized upon drawing, and possibly in situ and in real time using synchrotron facilities. In the case of 3D printed TP, chain orientation mechanisms were investigated by time-resolved SAXS/WAXS for PLA/hydroxyapatite nanocomposite upon cyclic tensile/thermal solicitations [67]. The latter induced orientation/relaxation of the amorphous chain characterized by the strain level of the amorphous peak position. With the increasing stress level at 70 °C, amorphous peak strain increased (decrease of the distance between amorphous chain [63], whereas (1) cooling from 70 °C to 22 °C at the maximum stress level, (2) stress unloading at 22 °C, and (3) heating without stress up to 70 °C led to a decrease in amorphous peak strain (increase in the distance between amorphous chain) (Figure 7b). These previous works proved the applicability of the SAXS/WAXS methods to studying molecular orientation in 3D printed TP, but did not reveal the full picture of molecular behavior (limited imposed stress/strain, little or no information about crystalline phase, no investigation of the initial chain-orientation anisotropy). 

### 2.5. Research to Be Tackled

Most of the previous studies related the macroscopic thermomechanical properties of 3D printed CFRTPCs with the material design and microstructure to tentatively improve interface bonding and reduce the corresponding defect formation, which is considered their weakest point. Attention has been mainly focused on reducing porosities induced by the different interfaces and on increasing polymer interchain diffusion between printed lines. Based on recent works [12,16], with the current porosity fraction of CFRTPCs being in the range of 5–10 vol%, it is not yet acceptable for aerospace application, which requires less than 1 vol% of porosity, but is near the acceptable limit for other applications, including automotive, i.e., 5 vol% [68]. Some research activities to be tackled were identified to further stimulate the development of 3D printed CFRTPCs. 

A specific challenge for the development of FFF process and the performance of printed parts is related to temperature control of the printing line since it has a direct influence on TP crystallization behavior. More particularly, this temperature significantly influences the interface temperature between the current printing line and the previously deposited layer and therefore controls the interchain diffusion phenomenon [25] that governs the interlayer adhesion [42]. This is why the addition of in situ pre-heating devices to plain FFF printers was reported by many authors (see Section 2.3). The ability of laser systems to deliver a given heat quantity on a given surface is definitely an asset compared to other kinds of devices and can explain the improved mechanical performance reported by some studies for neat TPs [43] or CFRTPCs [21]. In addition, better thermal management of the printing process in FFF can be obtained through a combination of thermal modelling and an experimental approach and may contribute to the printing of specimens with improved mechanical performance [69].

An important aspect of 3D printed CFRTPC development is the improvement of the microscale interface between the continuous fibers and the TP matrix. The development of appropriate sizing, dedicated surface treatment, or the impregnation of fibers in a dedicated resin is expected to be further investigated shortly. The coupling technology between the fibers and the TP matrix should address both the bonding efficiency and the deformability needed for the printing, which appears very challenging. 

Furthermore, the molecular aspects of the TP matrix in 3D printed CFRTPCs has been considerably neglected. Indeed, the extrusion induces a heterogeneous orientation state of the matrix within the printed line. The latter is expected to drastically influence molecular behavior during the solidification (crystallization) and the deformation (orientation) of the matrix. 

The rapid cooling of the melted TP matrix against the previously deposited layer at the substrate temperature (generally in the range of 60–120 °C) engenders the isothermal crystallization of the TP matrix, influencing nano-interface bonding and consequently the mechanical properties of the material. In regions of high initial orientation, fast crystallization is expected, and inversely. Isothermal crystallization kinetics of the TP matrix have been rarely studied by a systematic investigation in the case of 3D printed materials. 

When a strain is applied to a 3D printed CFRTP component, the polymer matrix is solicited and may orient and transmit stress to the continuous fiber. In regions of high initial orientation, further orientation induced by the imposed strain may be limited, and inversely. To our best knowledge, strain-induced orientation mechanisms of the TP matrix have not been investigated in detail. 

Obviously, TP matrix crystallization kinetics and orientation mechanisms should be further investigated. The challenge here relies on the initial structural heterogeneity of the TP matrix engendered by 3D printing (significant shearing by the extrusion nozzle and the presence of interfaces) that has to be taken into account. 

Better thermal management of the printing line process, further improvement of the fiber-TP matrix interface bonding and deformability, and further understanding of TP molecular aspects are highly desirable to develop the next generation of ultralightweight, mechanically durable, and smart FFF materials. 

## 3. Structure Health Monitoring 

### 3.1. Fiber Bragg Grating Sensors

**Figure 8:**
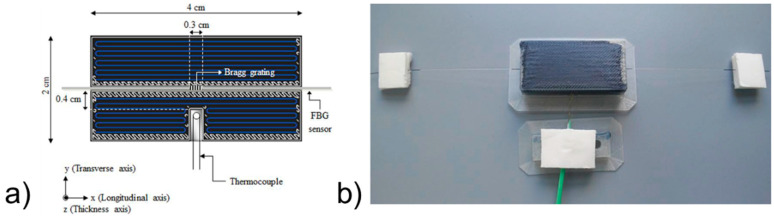
(**a**) Schematic of the midplane of a 0° fiber reinforcement orientation of a 3D printed CFRTPC with the location of the FBG sensor and the thermocouple, and (**b**) picture of a 3D printed CFRTPC specimen containing an embedded FBG sensor and a thermocouple [71] (copyright (2019), with permission from Elsevier).

**Figure 9:**
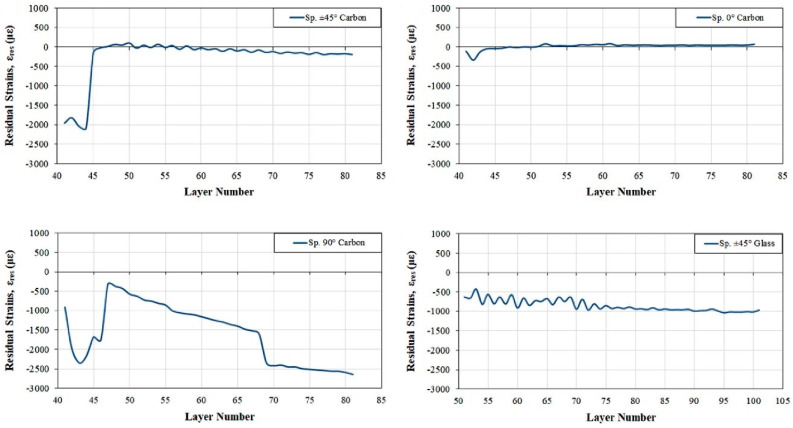
Effect of 3D printed CFRTPC’s fiber orientation and type on process-generated residual strains [71] (copyright (2019), with permission from Elsevier).

**Figure 10:**
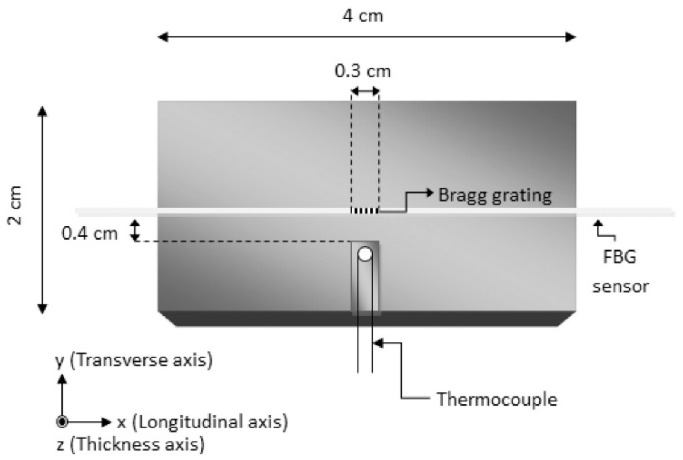
Schematic of a 3D printed neat TP with the location of the FBG sensor and the thermocouple [72] (copyright (2016), with permission from Elsevier).

**Figure 11:**
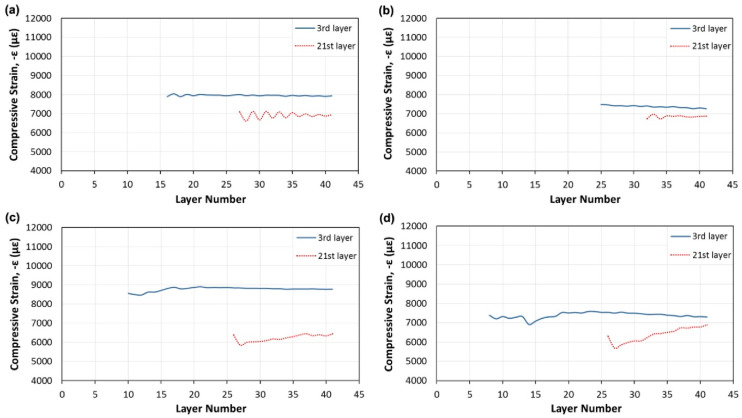
In situ measurements of the compressive strain of 3D printed neat TP as a function of layer number for horizontally (**a**,**b**) and perpendicularly (**c**,**d**) positioned specimens on the print bed, where the FBG sensors are longitudinally or transversely embedded, respectively [72] (copyright (2016), with permission from Elsevier).

Fiber Bragg grating (FBG)-based sensors have been used for the last three decades in various applications because of their environmental stability, light weight, small dimensions, high sensitivity, multiplexing ability, and immunity to electromagnetic interference [70]. They hence appear suitable to be integrated into CFRTPC materials as strain sensors. Nonetheless, this type of sensor integration into CFRTPCs is seldom reported, and there is room for further investigation. Kousiatza et al. [71] reported the embedment of FBG sensors in continuous carbon or glass fiber-reinforced Nylon composite specimens during the FFF printing process. The carbon fiber-reinforced specimens were printed with three different fiber orientations (±45°, 0°, and 90°), whereas the glass fiber-reinforced specimen was designed with fibers oriented only at ±45°. It is worthwhile to mention that the printed specimens displayed the same length and width, but the glass fiber-reinforced composites were deposited with a higher number of layers. The bottom part of each specimen was printed until the system reaches the midplane, at which point the deposition process was paused to allow an optical fiber sensor and a K-type thermocouple to be manually embedded (Figure 8a). Thereafter, the printing process was resumed, and the temperature and residual strains were monitored until the end of the printing process, as shown in Figure 8b. Afterwards, measurements were further recorded after the 3D printed CFRTPC specimen was removed from the print bed. In addition, based on the recorded FBG wavelengths under thermal loads, the thermal expansion coefficients of each 3D printed CFRTP composite were also calculated within the relevant ranges of temperature. The interface between the FBG sensor and the CFRTPC material was observed by digital microscopy. The magnitude of the residual strains of FFF-fabricated composite samples generated during the 3D printing process was calculated. It was based on the peak wavelength values recorded by the embedded FBG sensors and was influenced by the fiber type and orientation, as revealed in Figure 9. These characteristics had similar effects on the temperature profiles generated by the process, as well as the measured coefficient of thermal expansion (CTE) values of the composite specimens. The greatest magnitude measured for post-fabrication compressive residual strain in a free-standing state, i.e., when the parts are fully detached from the print bed, was related to the specimens reinforced with carbon fibers oriented at 90° with a value equal to 3100.2 microstrains (με). Concerning the temperature variations during the sequential layer deposition recorded by the thermocouple, it was uniform in the case of 0° and 90° reinforcing fiber orientations and non-uniform when the reinforcement was oriented at ±45°. As might be expected, the fiber type had significant influence due to its thermal properties. In another work by the same authors [72], a similar approach was reported, but on neat TP printed samples instead of 3D printed CFRTPCs. More precisely, in situ monitoring of 3D printed neat TP acrylonitrile butadiene styrene (ABS) was performed by means of the integrated FBG sensors and thermocouples enclosed by different layers (Figure 10). Equally important, the dependence of the magnitude of the generated residual strains on the specimen’s position on the print bed was also investigated. The real-time compressive strain magnitudes obtained from the Bragg peak wavelength values at the bottom part of the specimen were smaller than the average ones in the upper part, as given in Figure 11. Changes were only detectable while the deposited first layers were fully solidified. The greatest magnitude measured for post-fabrication compressive residual strain in a free-standing state corresponded to the third layer while the specimen was horizontally positioned on the print bed with longitudinally embedded FBGs with a value equal to 7384 microstrains (με). In that study, the temperature profiles during the 3D printing process were not only derived from the thermocouples but also calculated from the peak wavelength values of the FBG sensors by neglecting the solidification residual strains and knowing the initial reference temperature.

In the case of composites manufactured by conventional methods, FBG sensors are currently used in aeronautics, space, defense, nuclear power, transportation, and civil engineering for smart processing, quality control, health monitoring (defect detection), and control of the reinforcement of old structures [73]. The important spread of such sensors in composite applications is due to their high reliability. Their utilization in the case of 3D printed composites or polymers is still in its infancy due to the recent FFF technology. Recently, FBG sensors were successfully used to monitor the residual stresses during the FFF process of a PLA matrix, enabling a better understanding of the resulting structural properties [74]. 

### 3.2. Strain Gauge Sensors

In addition to FBG sensors, other sensors exist to detect strain in structures during their utilization. The most conventional technology is the use of a strain gauge that can now be manufactured by 3D printing, giving rise to a significant number of designs. By using the 3D printing technology Aerosol Jet, it is possible to deposit conductive ink-containing nanoparticles directly onto a substrate followed by a thermal sintering procedure to stabilize the nanoparticles’ conductive path [75]. This printing technology enables us to print micrometer-sized conductive lines, and hence, millimeter-sized strain gauges are generally developed. In other studies, the strain gauge conductive path was simply processed by FFF using a conductive polymer containing conductive particles [76]. By using FFF technology, the strain gauge conductive path has millimeter-sized conductive lines, and hence, a centimeter-sized strain-gauge sensor results. Different studies were found showing the successful implementation of 3D printed strain sensors into 3D printed materials by FFF [76,77,78,79]. In [76], a strain gauge with the conductive paths made from a graphene-filled conductive filament was integrated into a 3D printed PLA specimen. When submitting the PLA specimen to a loading and unloading procedure, the strain gauge signal has to be calibrated due to the non-linear response of the sensor, the hysteresis effect, and the repeatability deviation that are the drawbacks of this kind of sensor due to the viscoelastic nature of the strain gauge base material and/or of printed ink [79]. The 3D printing of strain sensors is also conducted onto highly flexible bases (elastomeric materials) to detect large strain level [80,81]. These flexible strain sensors open the way to numerous applications as sensing soft structures (interactive robotics, human motion detection, personal health monitoring, etc.) [82]. More detail about all the possibilities of 3D printing to create sensors can be found in these two reviews [13,83]. 

### 3.3. Self-Sensing Technology

**Figure 12:**
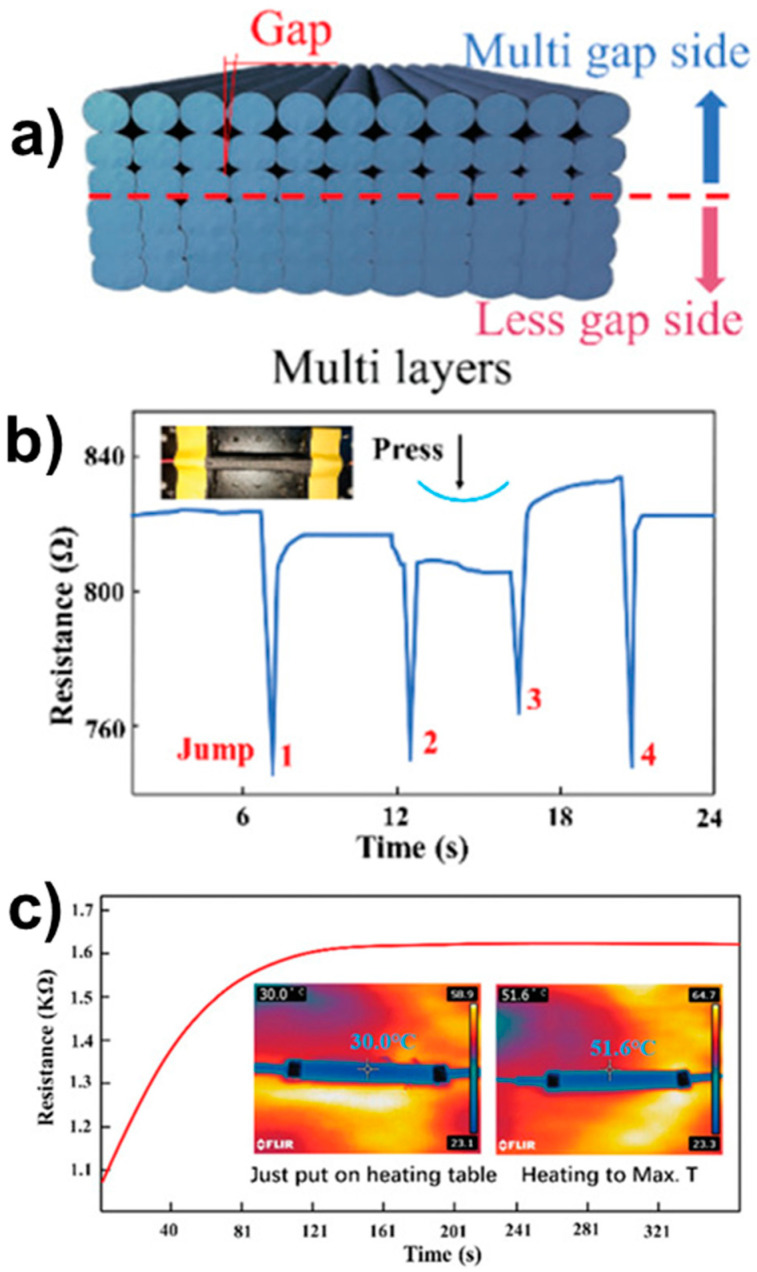
Self-sensing technology to detect strain and temperature variation in 3D printed PLA/carbon black composite: (**a**) material design showing gaps, (**b**) resistance variation when the composite is subjected to compression, and (**c**) resistance variation when the composite is subjected to a temperature increase from 30 °C to 52.6 °C [91] (copyright (2020), with permission from Wiley-VCH Verlag GmbH & Co).

An important technology developed for health monitoring is the so-called self-sensing technology, for which the 3D printed material is used to sense its structural state, acting as a strain sensor [84,85,86,87,88,89,90,91]. The 3D printing materials enabling such a self-sensing ability of the material is part of 4D printing technology [91]. The structure monitoring is based on measuring the electrical resistance of the materials, for which a certain level of electrical conductivity is ensured by conductive agents such as carbon black, graphene, carbon nanotubes, carbon continuous fiber, or copper wire leading to conducting paths. Some important aspects of this technology are the design of the conducting paths offering multiple possibilities thanks to FFF, and the data collection (from one conducting path or a network of conducting paths) and treatment to determine the variable of interest (strain, damage onset, etc.) [13]. To detect strain variation upon mechanical stress, the material design can include gaps or vacant paths (Figure 12a) that close when compressed or reopen when unloaded, leading to variation of the resistance (Figure 12b). The resistance also varies as a function of the material temperature (Figure 12c). In [92], a smart continuous carbon fiber tow-sensing grid was designed and integrated into a 3D printed thermoplastic matrix. The advantage of this system is that it provides a 2D strain field of the material that appears suitable to obtain local information, and hence, more precise sensing and detection of potential damage. As suggested in [91], the application range of this technology includes intelligent devices (biomedical instruments, human-machine interaction systems, protection devices, robots, etc.), but also conventional applications such as a bikes (Limburg Bike project, Brightlands Materials Center (the Netherlands), [93]) and helmets ([89], with the use of another 3D printing technology—photocuring 3D printing) with the detection of damage to improve safety. It is important to mention that mainly prototypes are currently being developed using self-sensing technology. 

### 3.4. Research to Be Tackled

Monitoring strain in 3D printed neat polymer and CFRTPCs during their utilization can be done based on the three promising technologies reported in the previous sections. Each of them has drawbacks and advantages that can be briefly discussed below. 

FBG sensors appear highly reliable and their signal treatment is well controlled. They have been used for decades in numerous industries, including aeronautics, but it is important to mention some important drawbacks of this technology. They are in principle more suitable for large-scale structures such as airplane wings [94] than for small-scale applications due to their geometry (continuous fiber to embed) and size (diameter of 250 µm [94]). As a component to embed in a structure, FBG sensors may negatively affect the mechanical properties of the host structure. A 2D strain field cannot be determined with only one FBG sensor, meaning that to precisely determine a damaged area, a network of multiple FBG sensors is needed based on a multiplexed FBG array configuration [95]. Again, such a sensor configuration is suitable for large-scale applications. Last, the performance of the FBG sensors drastically depends on the interfacial bonding between the FBG sensor coating and the host material, and the strain transfer between these two counterparts [94,96]. This coating facilitates the bending of the optical fiber and protects it against corrosion. It is generally made of a polymer such as polydimethylsiloxane, polyimide, or acrylate [97], or a polymer-based composite [94]. It was proven by modelling that the longer the bonded length is and the stiffer the coatings are, the more strain is transferred to the optical fiber [96]. To our best knowledge, the interfacial bonding between the FBG coating and the host 3D printed polymer of CFRTPCs has not been optimized yet, considering that the host polymer matrix is generally different from the FBG coating polymer. This would rely on developing coupling methodologies between two immiscible polymers. 

Strain gauges printed in 3D and integrated into 3D printed polymers or CFRTPCs are a new technology that provides freedom in design and dimensions. Furthermore, the polymer base of the conductive lines can be of the same nature as the host polymer structure, limiting interfacial issues. Nevertheless, some drawbacks have to be highlighted. The strain gauge can only measure strain along the major axis, meaning that a 2D strain field cannot be determined. More importantly, the recorded signal from strain gauge printed onto a polymeric base suffers from non-linearity, hysteresis, and repeatability deviation, implying drastic corrections of the signal to accurately assess strain [76,98]. Current developments of strain-gauge technology involve the miniaturization of the sensor facilitating 2D strain-field assessment [82] and improvement of the sensor device design to reduce the viscoelastic effects on the signal, and hence, reduce the correction severity. 

Structure health monitoring by self-sensing has the advantage of limiting/avoiding the embedding of a sensor, and hence, does not involve additional interfaces as structural weak points. This technology, such as the 3D printing of strain gauge, enables important freedom concerning the conductive path design and has been proven to permit the determination of a 2D strain field. Due to the presence of a polymeric matrix, the detected signal upon deformation may also suffer from viscoelastic effects, as highlighted for strain-gauge technology. Local strain measurements may help to detect any damage in the 3D printed neat polymer or CFRTPC. To go further, it would be relevant to identify the type of damage (matrix debonding, fiber rupture, opening of existing pores at the different interfaces, etc.) based on electrical resistivity measurements. It is hypothesized that each damage phenomenon has a unique signature that may be detected by a 2D strain field. 

Whatever the type of sensing technology, to our best knowledge, little attention has been focused on the mechanical durability of health monitoring technologies and the identification of the underlying mechanisms. Yet such a study may help to further improve sensing technology. The influence of thermal and UV aging on 3D printed health monitoring functionality is also a subject of high interest. 

## 4. Technology Landscape and Future Trends 

Continuous fiber-reinforced polymers are currently a top trend in 3D printing developments. This is connected to the potential of those materials to match conventional composite mechanical properties. FFF of CFRTPCs has only been accessible recently (2014). Thanks to their effective reinforcement an increasing number of applications are expected, especially in the automotive and soon in the aeronautic sectors. Even though a considerable amount of research work has been done on FFF, the number of scientific publications studying printed CFRTPCs remains scarce. Yet some recent reviews covered the advances in FFF of CFRTPC materials and processes [4,99,100].

In addition, a significant number of patents have been issued, demonstrating the growing interest in 3D printed fiber-reinforced composites. It should be noted that due to a growing number of patents related to 3D printing technology, patent offices introduced in 2015 a new cooperative patent classification (CPC) category (B33Y) facilitating patent searching and reviewing. Further subclasses were also created, including process, accessories, auxiliary operations, data acquisition, raw materials, and products. However, to the best of our knowledge, no reference has addressed the intellectual property (IP) aspects concerning FFF adapted to CFRTPC printing (i.e., from a raw materials, printing processes, accessories, or products perspective).Although it is important to cover IP aspects of a new technology in detail when reviewing its recent developments, our approach only addresses general trends observed on a selection of 398 patent families around the lines of FFF-based technologies for printing composite materials. With this view, various patent databases (Questel-Orbit, Espacenet, Patentscope) were consulted in February 2021 using various complementary keywords, searching queries, and refinement methods. 

**Figure 13:**
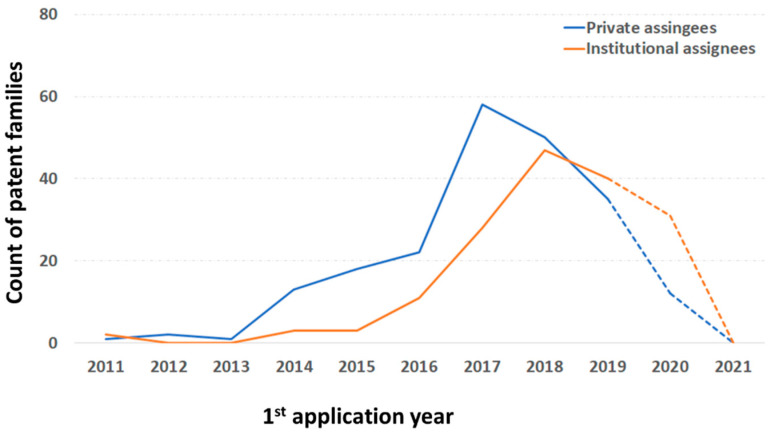
Count of patent families vs. first patent application year around the lines of FFF adapted to CFRTPC printing (including all aspects: process, accessories, auxiliary operations, data acquisition, raw materials, and products) in February 2021. Over the last 20 years, private assignees contributed to 213 patent families, whereas institutional assignees made up 185 patent families.

**Figure 14:**
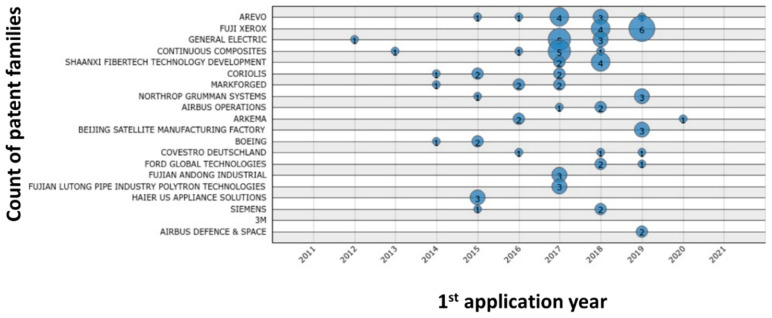
Count of patent families over the last 10 years for the 20 most active private assignees in FFF adapted to CFRTPC printing in February 2021.

**Figure 15:**
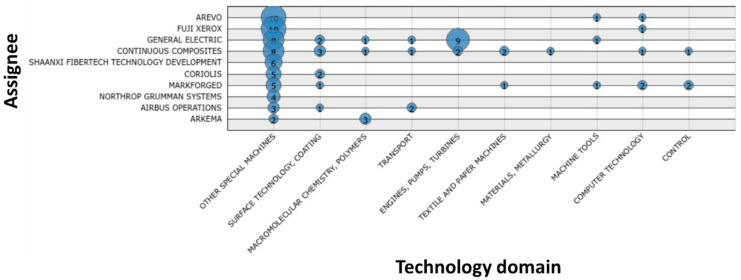
Main technology domains covered by the 10 largest private assignees in FFF adapted to CFRTPC printing in February 2021.

**Figure 16:**
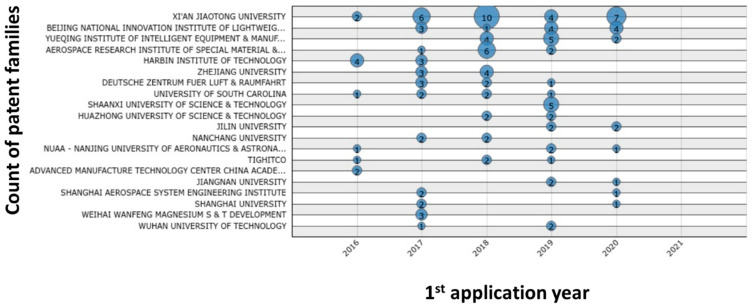
Count of patent families for the 20 largest institutional assignees in FFF adapted to CFRTPC printing in February 2021.

**Figure 17:**
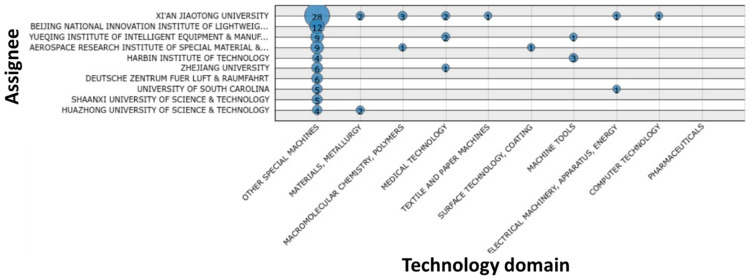
Main technology domains covered by the 10 largest institutional assignees in FFF adapted to CFRTPC printing in February 2021.

The quantitative analysis of selected patents (Figure 13, Figure 14, Figure 15, Figure 16 and Figure 17) indicates that the recent interest in fiber-reinforced composite printing via FFF dates back to 2013, when the number of initial patent applications started to increase significantly. As pictured in Figure 13, both private and institutional assignees were and remain equally active in patenting this technology. Figure 14 and Figure 16 list the 40 most active actors and show respectively that although a large variety of countries is represented in the private sector, Chinese assignees represent most of the institutional sector (120 out of 185 in total). Furthermore, Figure 15 and Figure 17 interestingly summarize the various technology domains covered by the main assignees. In particular, these figures show that both private and institutional assignees focused their innovative activities predominantly on the printing processes so far. This suggests that this technology is still in an early stage of maturation and that an increasing number of patents focusing on applications should be issued in the coming years. A huge potential for technical invention based on CFRTPCs printed by FFF is anticipated. However, a more qualitative analysis should be performed to refine the technology domains of interest for the authors, such as the automotive and aeronautics ones. 

FFF of CFRTPCs is driven by important opportunities in advanced applications. During the last seven years, many companies have launched their own solution. Most of the private companies are in the United States and Europe: Markforged (Watertown, MA, USA), 9T Labs (Zurich, Switzerland), Anisoprint (Moscow, Russia), Desktop Metal (Burlington, MA, USA), APS Tech Solutions (Höchst, Austria), Arevo (Santa Clara, CA, USA), Thermwood Corporation (IN, USA), CEAD (Delft, Netherlands), Mantis Composites (San Luis Obispo, CA, USA), Continuous Composites (Coeur d’Alene, ID, USA), Electroimpact (Mukilteo, WA, USA), Ingersoll Machine Tools (Rockford, IL, USA), Moi Composites (Milan, Italy), and Orbital Composites (San Jose, CA, USA). 

**Figure 18:**
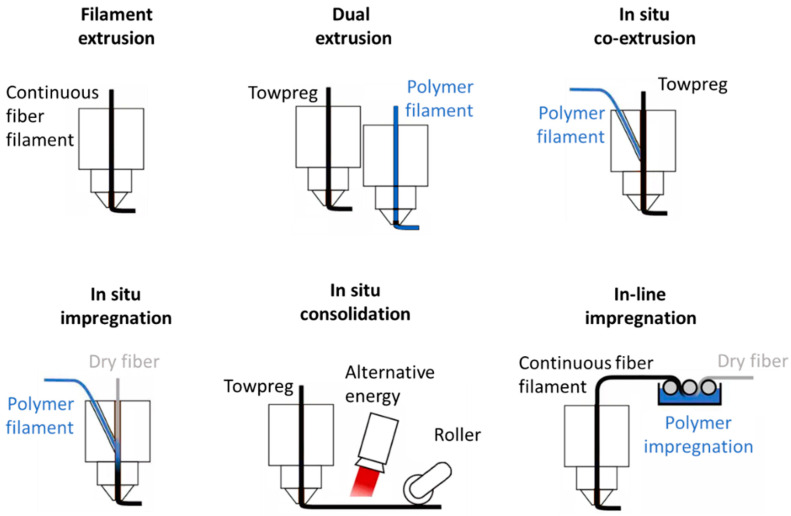
Main commercial of FFF processes for CFRTPC printing classified by increasing complexity, inspired by [116].

The main differences between commercial CFRTPC FFF printers are summarized in Figure 18 based on how and when the polymer matrix and the fiber are brought in contact. Table 1 classifies the companies according to the type and size of the printers they commercialize. Compared to other 3D printing methods, FFF offers many advantages, including cost-effectiveness and technical simplicity. However, for all CFRTPC parts printed by FFF, the main challenge is currently characterized by entrained air in the printed composite parts engendering the interfacial issues. Processes using already prepared continuous fibers and towpreg both present the advantage of dedicating an efficient manufacturing step to reduce and eventually eliminate air content. However, the FFF printing step can still bring some level of porosity, suggesting the need for further optimizations.

The filament extrusion process (or continuous fiber-reinforced thermoplastic filament extrusion process) stands for printers using readily made fiber-reinforced thermoplastic filaments, which are then simply heated during extrusion before being deposited [101]. This process is quite convenient, since it splits the complexity of the filament preparation from its printing, thus allowing greater control over each step. To material scientists, it offers the opportunity to easily evaluate (provided printers are equipped with an open software) various polymer matrix/towpreg combinations.

The dual extrusion process combines two printing heads: The reinforcing fiber towpreg and the thermoplastic resin filament are separately extruded onto a common printing plate [102,103,104]. It has been shown that printed parts obtained with this method generally present less porosity than the previous process. However, potential limitations can be anticipated due in part to both the design and poor infiltration of the fiber in the polymer [2,105].

The in situ co-extrusion process consists of supplying the polymer matrix and a towpreg separately to the print head. Then heated commercial towpregs or tapes are impregnated by a polymer matrix by co-extrusion [59,106]. The obtained continuous fiber-reinforced thermoplastic filament is then deposited. The polymer matrix is generally identical to that of the towpreg. However, in the case of Anisoprint’s process, the co-extruded polymer matrix is a thermoplastic, whereas the towpreg matrix is a thermoset [107,108,109]. Further polymer matrices should be available soon, increasing the spectra of application of this method.

In the case of the in situ impregnation process, dry fibers are fed into the printer head and then impregnated by a heated polymer matrix by co-extrusion before deposition [110]. The commercial versions of this process are dedicated to large-parts printing. The main advantage of this approach is to combine all steps in a single process using widely available and low-cost components such a continuous fiber and polymer filaments. It also allows a better control of the composite part volume fraction by playing on the component flowrates. However, despite being also reported as a rapid process, studies showed parts could contain defects [111,112]. 

The in situ consolidation process stands for a scaled-down version of a thermoplastic automated fiber-placement process [113]. Readily made towpreg or prepreg tapes are consolidated during and after deposition using various alternative energies. The reported commercial versions of this process are currently dedicated to large-parts printing. A melt pump and a pressure roller are also reported to apply mechanical consolidation after deposition. The reduction in printed part porosity associated with the use of low-pressure processing conditions during FFF printing has been reported to increase the interlaminar adhesion of various composite systems [114,115]. 

In-line impregnation is the most complex approach. The fiber is treated and impregnated by the polymer matrix before being directly transported into a hot print head and eventually deposited. Although it gives the best flexibility to the skilled users when evaluating various components (polymer matrix, interfacial treatment, continuous fiber), the approach requires that multiple manufacturing steps occur simultaneously, which makes optimization difficult. In addition, commercially, only large industrial printers are reported. 

As a conclusion, when looking at the diversity of processing techniques available, one important suggestion to select a continuous fiber reinforced FFF printer for research activities could be to favor the flexibility of the system (starting with an open software). As material engineers, the authors particularly favor lab-scale processing equipment allowing proprietary polymer formulations, filaments and towpreg to be manufactured easily. Eventually, such processing flexibilities could open the way to faster developments and improved properties, and be beneficial to new methods for improving printing quality such as strain-sensor technologies. In addition, enabling the systematic combination of various online controls, such as the management of temperature and pressure (IR, laser, roller, etc.) together with online surface modification of the towpreg and polymer filament as outlined in Section 2.3. or even fiber fraction, could also be beneficial to the optimization of printed parts and access to significant property improvements.

Some perspectives of FFF-printed CFRTPCs were recently discussed in several contributions [99,117,118,119,120,121,122,123,124]. The following future trends can be highlighted:

(1) FFF technology will lead to re-evaluation of the material choice for well-defined applications, with a high potential to replace metals with 3D printed CFRTPCs.
(2) FFF will further involve multi-material printing by combining CFRTPCs with other materials (TP composites with short fibers, elastomers, shape memory polymers, etc.), opening the way for novel functionalities and hence, applications.
(3) Concerning sustainability, CFRTPCs will probably have to address circular economy principles (recycling and remanufacturing), and the use of continuous natural fibers will be boosted.
(4) To facilitate, optimize, and fasten the FFF process, machine learning and artificial intelligence will be commonly used to select the best set of printing variables, and material design will be facilitated by the utilization of cloud computing.
(5) The mass production of high-strength and lightweight FFF-printed CFRTPCs will accelerate reaching the same fabrication speed of plastic molded products.

## 5. Conclusions

This review highlights the structure optimization and health-monitoring strategies of 3D printed neat TPs and CFRTPCs by fused filament fabrication, from which the following research needs were identified:

(1) A better thermal management of printing is required to facilitate interchain diffusion and further improve interface bonding between printed lines.
(2) A significant structural weak point is the interface between the TP matrix and the reinforcing fibers. This interfacial bonding should exhibit enough adhesive strength to provide an efficient reinforcement, but at the same time has to provide a certain flexibility during the printing process. New bonding strategies have to be developed accordingly.
(3) Further investigations into the molecular aspects of the TP matrix are needed to better understand and optimize the mechanical properties of 3D printed neat TPs and CFRTPCs. In particular, TP matrix crystallization kinetics and orientation mechanisms have been little investigated to date.
(4) FBG sensors embedded in 3D printed materials for health monitoring present a polymer-based coating, the interfacial bonding between the FBG coating and the host 3D printed polymer of CFRTPCs has not been optimized yet.
(5) Self-sensing and strain-gauge technologies used to detect strain variation in 3D printed materials exhibit an electrical signal that is highly influenced by the viscoelastic nature of the printed ink path or polymer sensor base or material matrix. Those two technologies have to be further optimized through new designs of the sensor to limit the correction of the electric signal.
(6) More research is needed in the context of multiple and miniaturized sensors and/or the 2D network of conductive paths to assess a 2D strain field to enable the detection of the highest level of strain and the damaged area with enough precision.
(7) Health-monitoring technologies do not enable the identification of the type of damage.
(8) The durability and influence of thermal and UV aging on the different health-monitoring technologies have been little investigated in the literature.

The technology landscape of CFRTPCs fabricated by FFF reveals that patents are mainly focused on the printing process and raw material development, indicating that this technology is at an early stage of maturation and has huge potential. FFF offers a significant number of strategies to improve the material structure to optimize mechanical performance and monitor the structural health, making this technology highly innovative and promising to solve societal challenges. However, research should be conducted on printers presenting a high flexibility in terms of component range (polymer filament and towpreg). The 3D printed materials of the future will probably conjugate ultra-lightweight, high mechanical properties, and multiple and sensitive functionalities. In this context, mass production by 3D printing will be a reality for numerous applications.

## Figures and Tables

**Table 1:** Suppliers of commercial FFF adapted to CFRTPC printing.

	Filament Extrusion	Dual Extrusion	In Situ Co-Extrusion	In Situ Impregnation	In Situ Consolidation	In Line Impregnation
Desktop printer	APS tech solutions Markforged	Markforged 9T Labs	Anisoprint	-	Desktop metal	-
Industrial printer	-	Markforged	Anisoprint	-	-	-
Robot/cell hybrids	Mantis	-		-	Arevo	Moi Composites
Robot	-	-		Continuous Composites Orbital Composite Moi Composites	Electroimpact Thermwood Corporation	-

## Data Availability

No new data were created or analyzed in this study. Data sharing is not applicable to this article.
